# Heterogeneous photosensitization for water reuse in cellars: evaluation of silica, spongin, and chitosan as carrier material

**DOI:** 10.1007/s11356-023-31178-0

**Published:** 2023-12-23

**Authors:** Andreia D. Santos, Eduardo Pinho, Patrícia Reis, Rui C. Martins, Marta Gmurek, Anabela Nogueira, Sérgio Castro-Silva, Luís M. Castro, Rosa M. Quinta-Ferreira

**Affiliations:** 1 CIEPQPF – Chemical Engineering Processes and Forest Products Research Center, Department of Chemical Engineering, Faculty of Sciences and Technology, University of Coimbra, Rua Silvio Lima, 3030-790 Coimbra, Portugal; 2 Polytechnic Institute of Coimbra, Coimbra Institute of Engineering, Rua Pedro Nunes, Quinta da Nora, 3030-199 Coimbra, Portugal; 3 Department of Molecular Engineering, Faculty of Process and Environmental Engineering, Lodz University of Technology, Wolczanska 213, 90-924 Lodz, Poland; 4 Lda, Rua de Fundões, 151, 3700-121 São João da Madeira, Portugal

**Keywords:** AOP, Cellar wastewater, Heterogeneous photosensitization, Photooxidation, Phthalocyanine photosensitizer, Wastewater treatment, Water reuse

## Abstract

Photosensitization, a powerful oxidation reaction, offers significant potential for wastewater treatment in the context of industrial process water reuse. This environmentally friendly process can be crucial in reducing water consumption and industrial pollution. The ultimate goal is to complete process water reuse, creating a closed-loop system that preserves the inherent value of water resources. The photosensitized oxidation reaction hinges on three essential components: the photosensitizer, visible light, and oxygen. In this study, we assess the performance of three distinct materials—silica, chitosan, and spongin—as carrier materials for incorporating the phthalocyanine photosensitizer (ZnPcS_4_) in the heterogenous photosensitization process. Among the three materials under study, chitosan emerged as the standout performer in reactor hydrodynamic performance. In the photooxidation process, the photosensitizer ZnPcS_4_ exhibited notable efficacy, resulting in a significant reduction of approximately 20 to 30% in the remaining COD concentration of the cellar wastewater. Chitosan demonstrated exceptional hydrodynamic characteristics and displayed a favorable response to pH adjustments within the range of 8 to 10, outperforming the other two carrier materials. To further enhance the efficiency of continuous operation, exploring methods for mitigating photosensitizer bleaching within the reaction medium and investigating the impact of different pH values on the process optimization would be prudent.

## Introduction

Over the past century, the demand for drinking water has grown 600%, whereas its availability has been decreasing due to climatic changes and anthropogenic pollution (Hanasaki et al. 2013; Ray et al. 2020). Industries use water for various purposes, such as cleaning, cooling, or heating, for steam generation, and as raw material or solvent. Although the industry sector uses less than 10% of the total withdrawal volume, it significantly stresses the water resources due to wastewater discharges. The deteriorating water supply quality raised water purchase costs, leading government entities to make stricter environmental standards and legislation. It forces industries to adapt and search for technologies that enable sustainable and efficient water usage (Ioannou et al. 2015; Rodrigues et al. 2022).

Wine production, in particular, generates a significant amount of wastewater, typically 2 L of wastewater and 0.5 kg of solid residue per liter of wine produced. Furthermore, the process requires considerable water, estimated at 1 to 4 L per liter of wine (Rodrigues et al. 2022). Effectively monitoring and managing water usage and wastewater production minimizes environmental impacts and ensures sustainable wine production practices (Zacharof 2017).

According to the International Organization of Vine and Wine, global wine production for 2022 is estimated to reach 260 million hectoliters, with Portugal anticipated to contribute 6.7 million hectoliters to the total (International Organisation of Vine and Wine 2022). Suggesting that during the 2022 harvest season, between 6.7 to 26.8 million hectoliters of water were consumed in Portugal and up to 13.4 million hectoliters of acidic, biodegradable, and phytotoxic wastewater with high organic matter were produced (Mosse et al. 2011). Reclaiming and reusing this wastewater could significantly reduce the water footprint of the wine-making industry.

There has been a significant focus on wastewater treatment aimed at implementing cost-effective and sustainable waste management strategies following Circular Economy guidelines, such as those outlined in Green Deal Europe and European Commission initiatives (Cañadas et al. 2022; European Commission 2019; Zacharof 2017). Researchers have found that extracting valuable platform chemicals, biofuels, heat and energy, and antioxidants from wastewater is possible. Due to its biodegradable nature, biological treatments, such as anaerobic and aerobic processes, have been employed (Bolzonella et al. 2019; Kumar et al. 2009; Miklas et al. 2022). Additionally, physicochemical techniques have been utilized, including precipitation, sedimentation, coagulation, electrocoagulation, advanced oxidation processes (AOPs), and membrane treatments. However, combining these processes is necessary to increase the efficiency of removing recalcitrant organic compounds and ecotoxicity while reducing investment and operational costs (Davididou & Frontistis 2021; Ioannou et al. 2015; Martins et al. 2009).

Apart from energy generation and the production of value-added products, treated wastewater reuse, usually reclaimed water, is a critical component of the circular economy in industries (Kundu et al. 2022). Treated wastewater is primarily used in agriculture for fertigation, which involves the application of fertilizer solutions to crops using irrigation water, typically through drip systems or micro-sprinklers (Obreza et al. 2020). This practice has proven to be a sustainable resource that can improve crop production and reduce the need for inorganic/synthetic fertilizers (Etchebarne et al. 2019; Kundu et al. 2022; Milani et al. 2020). However, industrial applications are also a viable alternative, as the treated wastewater follows the guidelines established by the European Union (European Union 2022) and regulated in Portugal by Law-Decree 119/2019 of August 21st (Decreto-Lei n.o 119/2019, de 21 de Agosto 2019).

Reclaimed water that undergoes tertiary treatment with disinfection can meet regulations and be used for various purposes, making it a valuable resource for industries, agriculture, and ecosystems. For example, treated water can be used in industrial processes, such as cooling and boiler feed water, reducing water demand and decreasing the environmental impact of industrial water use. Economic advantages for industries will also be relevant. Additionally, reclaimed water can be released into waterbodies to support aquatic ecosystems and maintain ecological health. These multiple applications demonstrate the versatility and importance of treated wastewater as a sustainable water resource. (Monte & Cunha 2021; Morseletto et al. 2022).

Conventionally, water disinfection for reuse is typically achieved through aggressive chemicals or physiochemical methods, such as chlorination, ozonation, or membrane filtration. However, these methods may produce harmful by-products or require significant energy consumption.

Solar-driven advanced oxidation processes (AOPs), such as photosensitized oxidation (PO), have emerged as promising and sustainable solutions for wastewater treatment (Valkov et al. 2018). Reactive oxygen species can be generated using photosensitizers and solar light, such as singlet oxygen (^1^O_2_), which can effectively degrade and remove organic matter and pathogens from wastewater. This approach proves to be successful in addressing a wide range of pollutants, including toxic organic compounds and microorganisms, while minimizing the use of harmful chemicals and reducing energy consumption. (Pandey et al. 2021; Vivar et al. 2021; Zhang et al. 2018).

## Theoretical background

### Photosensitization process

**Fig. 1:**
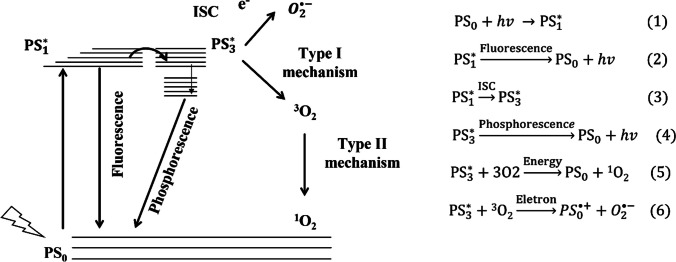
Jablonski diagram (adapted from Thandu et al., (2015) copyright license: CC BY 3.0) and the chemical reaction involved in the process

Photosensitization is when a molecule undergoes photochemical or photophysical changes when another molecule, called a photosensitizer, absorbs initial radiation. The photosensitized oxidation reaction requires three main components: a photosensitizer (PS), visible light, and oxygen (Baptista et al. 2017; Michelin & Hoffmann 2018). These reactions entail two mechanisms that can occur separately or simultaneously, aimed to oxidize the mixture’s pollutants. The type I mechanism involves a chain of redox reactions derived from an electron transfer. The second mechanism, known as type II, leads to the formation of singlet oxygen (^1^O_2_), a reactive oxygen species (ROS). ROS are chemical molecules and derivatives of molecular oxygen responsible for the oxidative degradation of contaminants (Thandu et al. 2015). The two possible reaction mechanisms are presented in Fig. 1, following Eqs. 1 to 6, where the PS in the ground state (PS_0_) absorbs photons, getting excited to the singlet state (PS_1_). There may be a phenomenon called fluorescence in this state, where the energy previously absorbed by the PS is released, returning to the ground state, or inter-system crossing (ISC), leading to a triplet excited state (PS_3_). Next, this state may lose energy via phosphorescence, causing the PS to return to the ground state. The photosensitizer in the excited triplet state (PS_3_) possesses a longer lifetime than the singlet excited state and can react easily with molecular triplet oxygen, following Eqs. 1 to 6 (Thandu et al. 2015).

The PO process may occur in a homogeneous or heterogeneous phase, relying on the contact between the PS and the effluent. The PS is mixed in the effluent during the homogeneous reaction, occurring in situ. The homogenous PO process has been applied first, despite disadvantages related to the application of soluble PS and difficulties with its recovery. Therefore, the hypothesis of reuse is discarded, which attains high costs, making this technique unfeasible economically. Alternatively, insoluble PS might be used, yet these compounds tend to be less active and are subject to aggregation, leading to a significant decrease in activity (Gmurek et al. 2017a, b; Ribeiro 2014).

In the heterogeneous process, the PS is immobilized onto particles called carriers, which helps overcome the homogenous process’s main drawback. The first-time phenomenon of anchoring, or immobilization, was successfully done in 1963 by Merrifield (1963), who connected the first amino acid of a polypeptide chain to an insoluble solid polymer by covalent bonding. This complex facilitates the subsequent separation or purification of the peptide, which usually gets more difficult for larger chains. The polymer used for this purpose was polystyrene cross-linked with divinyl benzene, now called Merrifield Resin, which is currently one of the most widely used resins (Gmurek et al. 2012; Vaino & Janda 2000).

### State of the art

This idea of immobilization has been adopted for other purposes, including applying PO in heterogeneous reactions. Immobilizing the PS onto carriers facilitates PS removal from the reaction medium (Gmurek et al. 2012; Gmurek et al. 2017a, b). There has been a growing interest in using carriers of natural origin and renewable materials. Developing carriers that combine those conditions *and* simultaneously create a material capable of degrading bio-refractory substances will provide an economical and environmentally sustainable approach to PS utilization, allowing the subsequent closure of the water cycle in industries where its consumption is high.

Several materials have been studied as composites for dye or ions adsorption (Pooladi & Bazargan-Lari 2023; Wu et al. 2023), along as supports for immobilizing photosensitizers in wastewater treatment, including silane gel, bentonite, polyamide hydrogel, silica supports and resins, polymeric nanofibers, and polyurethane. Table 1 provides an overview of these materials and their application with different photosensitizers, such as organic dyes, porphyrins, and phthalocyanines.

**Table 1:** Immobilized photosensitizer used by several authors for different contaminants removal reported in the bibliography

	Carrier material	Photosensitizer	Contaminant	pH	Removal efficiency (%)	IC reuse	Radiation source
Gryglik et al. (2004)	Silane gel	Rose Bengal Methylene blue Chlorin-e6	2-Clorophenol	7	80 90 90	1	Xenon light bulb 100 W
Xiong et al. (2005)	Bentonite	AlPc	Phenol 4-Chlorophenol 4-Nitrophenol 2,4-Dichlorophenol 2,4,6-Trichlorophenol	12	100 100 65 100 100	4	Halogen light bulb 500 W
Gmurek et al. (2012)	Polyurethane	TPP	2-Chlorophenol	9	81	6	Xenon light bulb 300 W
Ronzani et al. (2013)	Silica gel	Rose Bengal DBTP-COOH ANT-COOH	α-Terpinene	-	100 100 100	4	16 × light bulb 8 W
Drozd et al. (2014)	Polyethyleneglycol	_p-T_HPP	Phenol	10	100	4	Xenon light bulb 75 W
Norman et al. (2016a, b)	Spongin	CuPc	Rhodamine B	-	75	-	Mercury light bulb 150 W
Gmurek et al. (2017a, b)	Chitosan	AlPcS_4_ ZnPcS_4_	2,4-Chlorophenol Benzhylparaben	10.8	10 70	8 10	Sodium light bulb 250 W
Norman et al. (2018)	Spongin	FePc	Phenol Chlorophenol Bisphenol A Fluorophenol	-	100	3 3 1 3	Mercury light bulb
Foszpańczyk et al. (2018)	Chitosan	AlPcS_4_	Methylparaben Ethylparaben Prophylparaben Butilparaben Benzhylparaben	9	75 78 79 85 95	10	Sunlight
Neves et al. (2019)	Silica gel	H2TF5PP	Metoprolol	7.5	60	-	Xenon light bulb 75 W Sunlight
Foszpańczyk et al. (2019)	Chitosan	AlPcS_4_	Phenol 2-Chlorophenol 2,4-Dichlorophenol 2,4,6-Trichlorophenol acid 4-Hydroxybenzoic acid 4-Hydroxybenzoic Methylparaben Benzilparaben 2-Phenyl phenol 3-Phenyl phenol	9	40 100 100 100 65 100 70 75 75 75	12	Sodium light bulb 600 W

In various studies, Gryglik et al. (2004) investigated the oxidation of 2-chlorophenol using photosensitizers immobilized in silane gel supports. At the same time, Xiong et al. (2005) developed bentonite supports immobilized with aluminum phthalocyanine to degrade phenolic compounds. Both studies showed promising results in the efficient degradation of pollutants. However, the stability of the photocatalysts was found to be an issue, as noted by Gryglik et al. (2004) after the first reuse of the photocatalysts. In another study, Pepe et al. (2005) developed silica supports and resins immobilized with different photosensitizers, testing their ability to degrade phenol. They found that complexes containing porphyrins and phthalocyanines had the shortest degradation times and suggested avoiding excessive photosensitizer immobilization to optimize efficiency (Pepe et al. 2005).

Neves et al. (2019) achieved covalent binding between the photosensitizer H_2_TF_5_PP and silica supports to develop photocatalysts. They studied the degradation of metoprolol11 through PO in both homogeneous and heterogeneous media. Under homogeneous conditions, a degradation efficiency of approximately 90% was observed after 12 h of reaction. Under heterogeneous conditions with agitation, a degradation efficiency of roughly 60% was observed after 12 h. These experiments were conducted using simulated radiation and sunlight. A base was gradually added to prevent the solution’s acidification and subsequent photocatalyst deactivation. However, the degradation rate dropped to 40% in the absence of agitation and basic addition.

In a separate study, Norman et al. (2016a, b) conducted a series of studies on the immobilization of different pigments in spongin supports. They achieved an adsorption efficiency of 74.5% for the immobilization of anthocyanin in spongin supports, demonstrating the viability of this technique for immobilizing other types of pigments.

The degradation of Rhodamine B using copper phthalocyanine (CuPc) immobilized in spongin supports, assisted by H_2_O_2_ and UV radiation, was also studied by Norman et al. (2016a, b). With exclusive use of PO and UV radiation, they achieved a degradation efficiency of 70% after 1 h, which increased to 90% by adding H_2_O_2_. Authors have also studied the immobilization of iron phthalocyanine (III) (FePc) in spongin supports and their use for the degradation of phenolic compounds. They found that, in the presence of H_2_O_2_ and UV radiation, the total degradation of pollutants could be achieved within an hour, and the particles could be used for at least 3 cycles without losing their degradation capacity (Norman et al. 2018).

The utilization of chitosan as a carrier material was first introduced by Moczek and Nowakowska (2007). They synthesized and examined chitosan supports for the immobilization of Rose Bengal, demonstrating its potential as an attractive and viable material for the future synthesis of supports for applications (Moczek & Nowakowska 2007).

Foszpańczyk et al. (2018) conducted a comparative study on paraben degradation and water disinfection using photocatalytic and photosensitized oxidation (PO). They discovered that only PO achieved complete disinfection due to the high toxicity of oxygen singlet towards bacteria. Paraben degradation was comparable in both methods, with photocatalytic oxidation somewhat superior. They used AlPcS_4_ immobilized in chitosan supports for photosensitive oxidation and photocatalysts doped with noble metals for photocatalytic oxidation. In another study, Foszpańczyk et al. (2019) conducted another survey on the PO process using chitosan supports to degrade ten phenolic compounds using only AlPcS_4_. They observed a degradation rate of approximately 50% among the compounds studied and investigated the longevity of the supports by reusing them for 12 reaction cycles. The degradation rate slightly decreased between the 5th and 12th cycles. The authors generally observed that the degradation rate of pollutants increases with increasing oxygen concentration, light intensity, and photosensitizer concentration. Additionally, alkaline conditions were found to promote the degradation process. However, the study also revealed that the reusability of the photocatalysts was limited, with a significant loss of activity observed after the first reuse, indicating the need for further improvement in the stability of these complexes.

PO has also been evaluated as an effective approach for wastewater decontamination (Bartolomeu et al. 2017; Du et al. 2020; Valkov et al. 2018). Bartolomeu et al. (2017) have studied the photoinactivation of *E.coli* and phenol removal in treated urban wastewater using Tetre-Py^+^-Me porphyrin as a PS under artificial white light (40 W.m^−2^). The authors proved that the used PS was effective in inactivating the bacteria and oxidizing phenol, concluding that the use of PO as a water disinfectant avoided the contamination of the receiving water with the already employed disinfection products, such as chlorine, and the potential of microorganic mutagenicity caused by UV. Similarly, Valkov et al. (2018) evaluated four PS (Rose Bengal sodium salt, Rose Bengal lactone, methylene blue, and hematoporphyrin) immobilized in polyethylene or polypropylene for water disinfection. Their results showed that the immobilized PS exhibited antibacterial activity and could be used for water disinfection.

All mentioned works show that PS is effective for phenol and paraben degradation. Nevertheless, the organic matter degradation is noted as effective or slightly reduced. This study aims to assess the performance of chitosan, silica, and spongin as carrier materials for heterogeneous photosensitization of winery wastewater to remove residual organic matter.

## Materials and methods

### Photosensitizer and carriers’ preparation

This study utilized three carrier materials: silica, chitosan, and *Hippospongia communis*. The carrier preparation varies with the used material. Silica spheres (*d* = 2.64 ± 0.15 mm) were dried at 105 °C for 24 h to remove residual water. The chitosan carriers or beads were prepared according to (Gmurek et al. 2018): a chitosan solution with a concentration of 60 g/L was mixed with 8% (v/v) acetic acid solution and left in the dark for 24 h. The chitosan beads (*d* = 3.34 ± 0.15 mm) were made by dropping the chitosan/acetic acid solution into a 10% (m/m) NaOH solution. After the beading process, the beads were washed with deionized water until neutral pH. The *Hippospongia communis* requires a cleaning process with deionized water before use. After the bath, it was submerged in a 3 M chloride acid solution for 72 h to dissolve residual calcium carbonate. After this process, the *Hippospongia communis* was dried at 50 °C for 24 h and cut into cube shapes with a 2 cm edge length.

### Photosensitizer

**Fig. 2:**
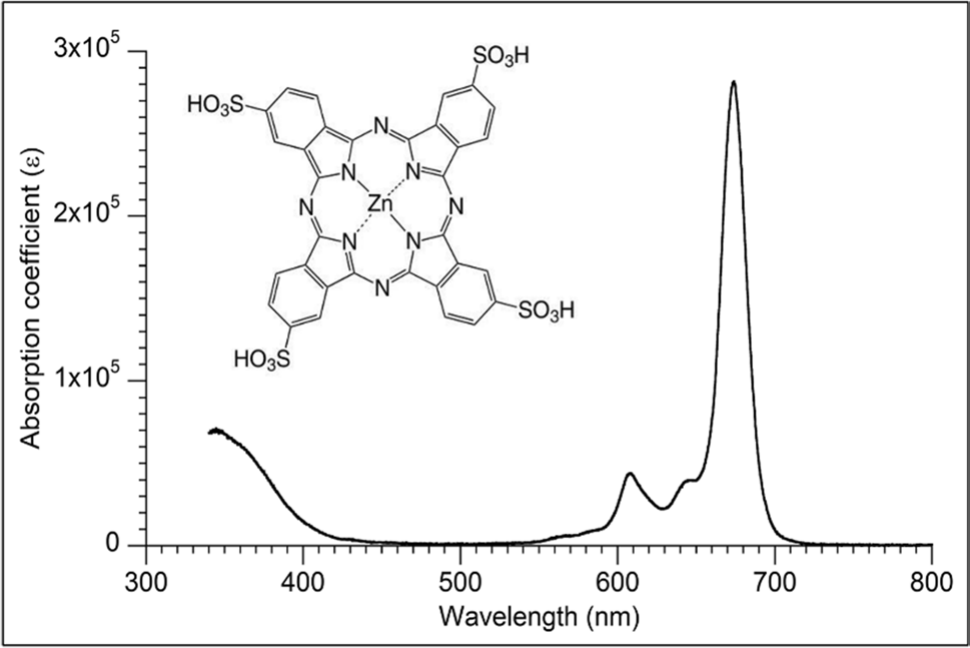
Zinc phthalocyanine tetrasulfonate (ZnPcS_4_) molecular structure and absorption spectrum

As a photosensitizer, Zn phthalocyanine tetrasulfonate (ZnPcS_4_) (Frontier Scientific, Newark, DE, USA) was used. The PS selection for this study was based on previous studies regarding this wastewater and different PS, as reported in Santos et al. (2023). Figure 2 shows the absorption spectrum of the ZnPcS4, available on the PhotochemCAD™ database (Lindsey et al. n.d.).

Phthalocyanines are aromatic and macrocyclic organic compounds widely used in photochemistry, such as zinc, aluminum, and titanium, form metal phthalocyanines when they react with solar radiation. The degree of sulfation affects the emission of energy by the photosensitizer and the excitation of molecular oxygen to singlet oxygen in wastewater. To prevent cluster formation, phthalocyanines must be monomeric to increase sunlight excitation efficiency. Therefore, it is crucial to synthesize soluble monomeric phthalocyanine complexes for water contact (Ion & Ion 2017; Tratnyek et al. 1994).

The effectiveness of photosensitizers is affected by the central ion’s nature. Heavy metal ions or paramagnetic metal ions enhance the photosensitizer’s yield as a triplet but reduce its lifetime in the singlet state. The photosensitizer in the triplet state initiates the photo-redox reaction, and a longer triplet state lifetime benefits the photosensitizer by increasing the likelihood of diffusion shocks with potential inhibitors. Organic compounds containing double bonds, such as phenolic compounds, are potential inhibitors that compete with the photosensitizer by absorbing solar radiation (Darwent et al. 1982).

### Immobilization process

After preparing the carriers, they were used to immobilize the photosensitizer (ZnPcS_4_) by adsorption. The silica photocatalyst was prepared in acetic acid, and PS bath at 90 ppm, and the selected concentration was set following previous studies (Santos et al. 2023). The immobilization process was finished once the silica reached a green-to-blue color. A similar approach was used for chitosan, whereas the ZnPcS_4_ was dissolved in water, keeping the same concentration. The carriers were left in the bath for 24 h in the absence of light. For *Hippospongia communis*, the immobilization process followed the one described by Norman et al. (2018), with an acidic bath of 100 ppm photosensitizer concentration prepared to promote adsorption. The carriers were left in the mixed bath for 6 h. After the carriers’ immobilization, they were washed to remove unabsorbed PS. Deionized water cleaned the immobilized carriers until no photosensitizer was detected.

### Analytical methods

Several analyses were conducted to evaluate the impact of the photosensitizer and the immobilization process on each carrier. Fourier-transform infrared spectroscopy (FTIR) analysis was carried out in the Perkin Elmer Spectrum Bx-2 to verify the success of the immobilization process. X-ray diffraction (XRD) for searching for potential structural alteration on the immobilized PS surface by employing the Bruker D8 Advance diffractometer. The Brunauer–Emmett–Teller (BET) was employed to determine the surface area of the carrier and the immobilized PS, using the surface analyser Micromeritics ASAP 2000. Scanning Electron Microscopy (SEM) analysis, performed with TESCAN VEGA 3 SBH, can evaluate the carrier’s structural characteristics before and after immobilization. Due to the humidity in the chitosan and the lack of drying technology that keeps the beads’ structure intact, SEM was not performed for this carrier.

Chemical Oxygen Demand (COD) was determined by employing the standard Closed Reflux Colorimetric Method 5220 D as established in the Standard Method (APHA et al. 2017).

The assessment of the PS concentration was performed via spectrophotometry. The absorbance of standard solutions with known PS concentrations was measured at 765 nm, and a calibration curve was created. The evaluation of the PS concentration in the bathwater allows access to the PS bleaching.

### Wastewater and experimental methodology

During this work, two types of effluents were evaluated: E1—from a cellar industry in the Ribatejo region of Portugal—and E2—from a wastewater treatment plant in the north region of Portugal. These wastewaters were collected from the treated wastewater storage tank of a conventional activated sludge wastewater treatment plant of the winery facilities and stored at a temperature of 4 °C. Both wastewaters presented low COD concentrations (< 40 mg O_2_/L).

A lab-scale glass reactor with a 250 mL volume was used, and the air was supplied with an air pump with a maximum flow of 4.5 L/min; solar light was used as the light source. During the experimental assays, an aliquot of 5 mL was removed to evaluate the reactional medium during the assay duration (120 min). All the assays were made in double, and all graphics show the average results and the corresponding standard deviation.

## Results and discussion

### Carrier characterization

**Fig. 3:**
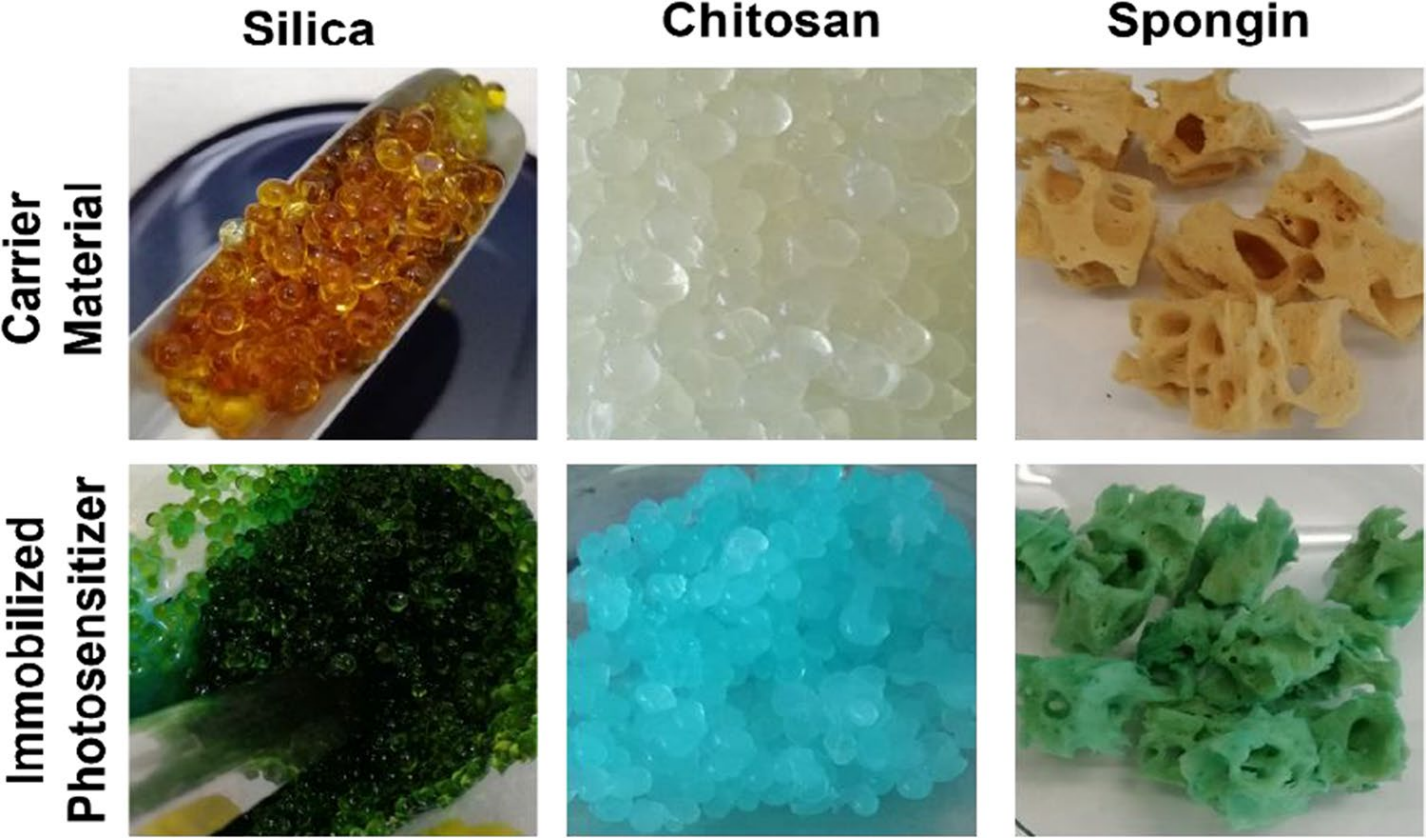
Color changing in the carrier material before and after immobilization

Due to the blue color of the PS, the adsorption into the carrier material was visible after the immobilization process, as shown in Fig. 3.

The success of the immobilization process can be confirmed through FTIR analysis once an increase in the absorption signal is observed in the range 3600–3100 cm^−1^. It is associated with the presence of the O–H and N–H groups in the sample (Foszpańczyk et al. 2019).

**Fig. 4:**
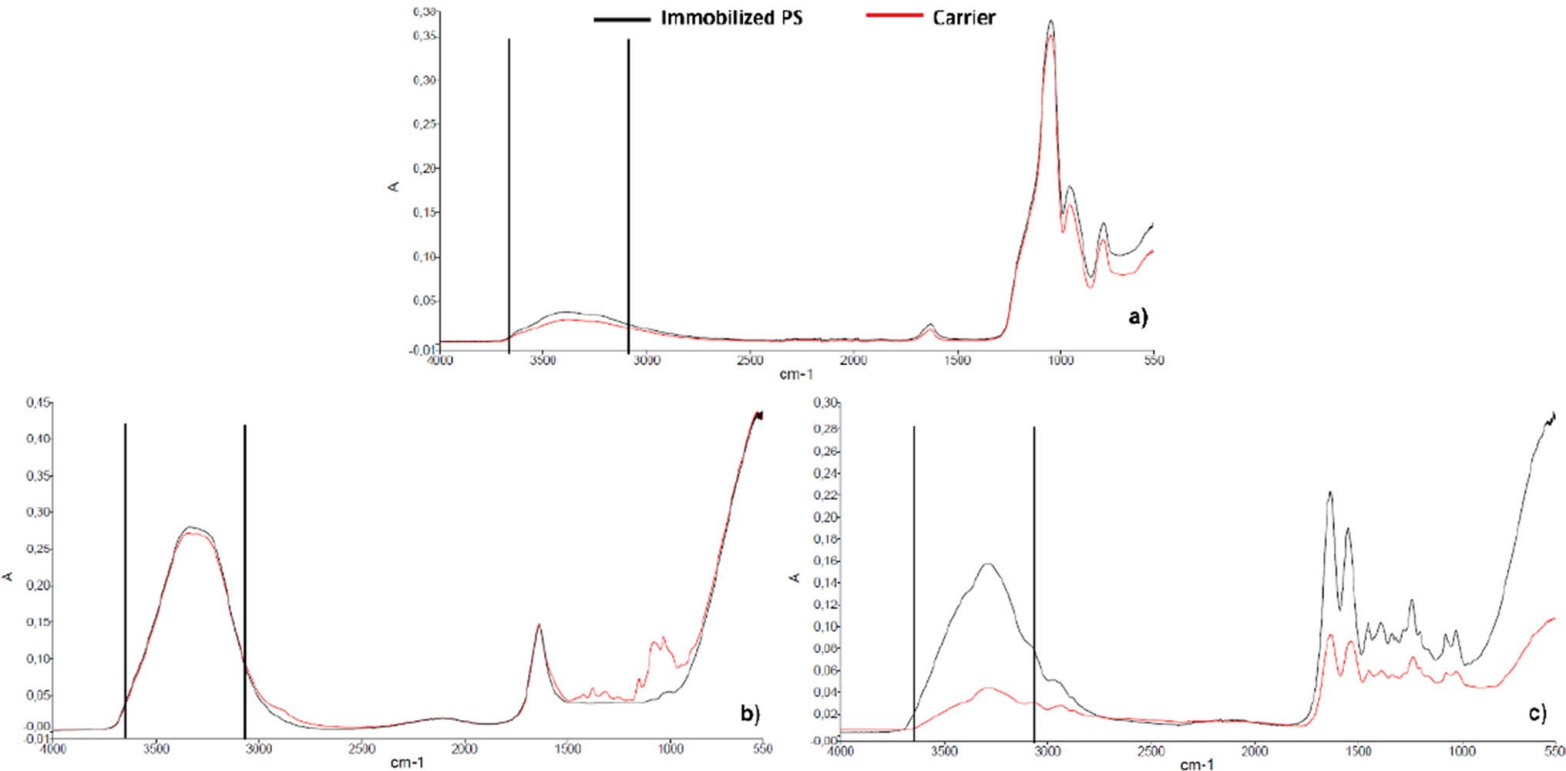
FTIR results for silica (**a**), chitosan (**b**), and spongin (**c**)

Figure 4 presents the FTIR results for all carriers and immobilized PS. It is verified that the immobilization process for the silica was not as effective as for the other carriers. The differences between before and after immobilization were confirmed, primarily in the 3600–3100 cm^−1^ range, indicating the presence of PS, as mentioned by Foszpańczyk et al. (2019). The increasing adsorption signal in that range after the immobilization is more evident in spongin than in chitosan and silica (Foszpańczyk et al. 2019).

**Fig. 5:**
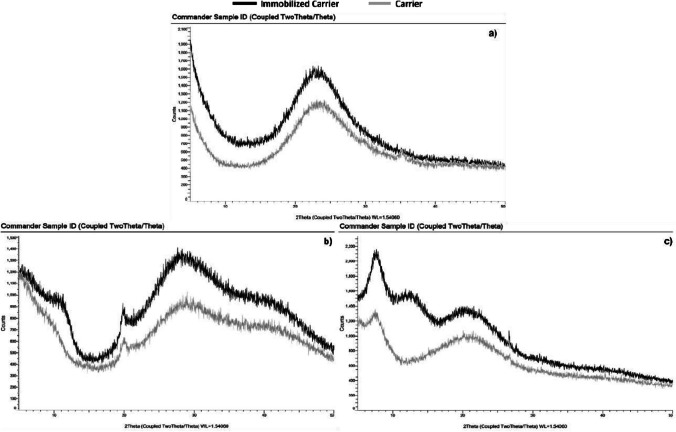
XRD results for silica (**a**), chitosan (**b**), and spongin (**c**) before and after immobilization

Figure 5 shows the XRD results for the silica, chitosan, and spongin as pristine carriers and with immobilized PS. Similar behavior is visible between the carriers and the photocatalysts for all carrier materials employed. The diffractogram of the immobilized PS indicates a higher X-ray intensity, which suggests that there has been a slight structural change in the material due to the immobilization process. This change may be due to the interaction between the PS and the carrier material and can potentially affect the properties and performance of the immobilized material. Furthermore, since there are no distinct peaks in the diffractograms, it is possible to conclude that the material under study is amorphous and free of crystalline structure (Chauhan 2014). Foszpańczyk et al.’s (2019) study revealed no structure alteration after immobilizing the PS in the chitosan carrier.

The results of the BET analysis presented in Table 2 show that the immobilized PS has a lower surface area than the pristine carrier. This decrease in surface area suggests that during the immobilization process, the PS occupies some of the pores of the carrier material. This finding indicates successful adherence of the PS to the carrier material, potentially leading to changes in the material’s properties and performance. Based on the results, the PS was successfully immobilized to all the carrier materials evaluated in the study.

**Table 2:** BET analysis for silica, chitosan, spongin carrier, and immobilized PS

	Surface area (m^2^/g)		
	Silica	Chitosan	Spongin
Carrier	542.47	75.25	55.24
Immobilized carrier	457.11	54.06	37.72

**Fig. 6:**
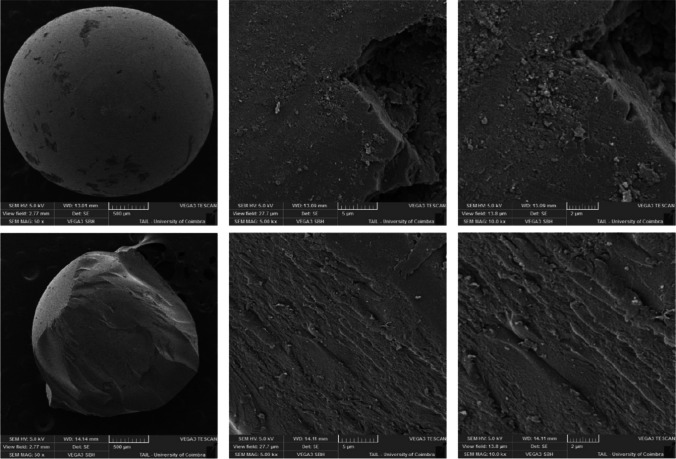
SEM images of the carrier (top) and immobilized carrier (bottom) for silica, magnified × 50, × 5000, and × 10,000

Three magnifications were used for the SEM analysis of silica: × 50, × 5000, and × 10,000. Figure 6 presents the SEM images for the silica carrier (top) and the photoactive new materials (bottom). The solvent on the surface of the carrier causes a noticeable structural difference between the carrier and immobilized PS. The higher ampliation shows minor damage on the smooth surface of the carrier after immobilization.

**Fig. 7:**
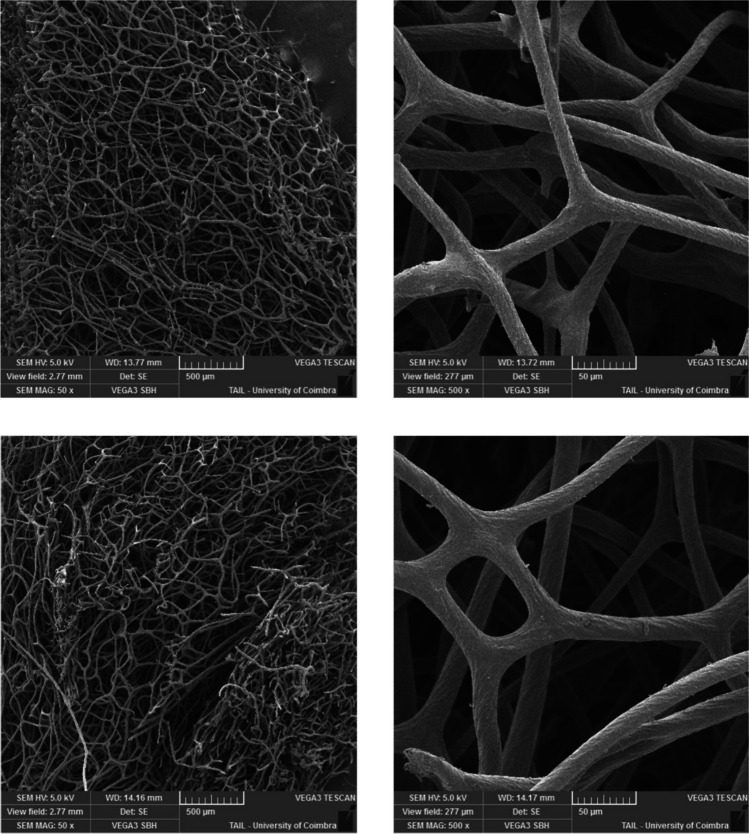
SEM images of the carrier (top) and immobilized carrier (bottom) for spongin, magnified × 50 and × 5000

The available techniques to dry the chitosan samples did not allow for the integrity of the particles. For that reason, it was impossible to perform the SEM analysis. Figure 7 shows the SEM images for the spongin carrier and immobilized PS magnified × 50 and × 500, where the spongin fibrous structure is visible. No significant changes are noticeable when comparing the carrier with the immobilized PS, except for the deposition of the PS in the carrier, which is visible in the photocatalyst images (bottom), where small PS particles can be seen on the spongin fibers. This deposition is more evident in the images at a magnification of × 50 than at × 500.

### Hydrodynamic performance

**Fig. 8:**
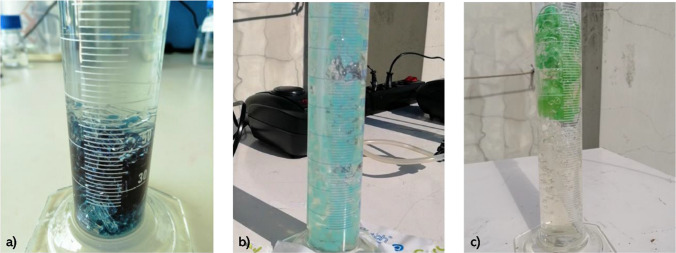
Hydrodynamic behavior of silica (**a**), chitosan (**b**), and spongin (**c**) immobilized carrier

Regarding the hydrodynamic behavior, it is observed that the silica-immobilized carrier is not ideal. These particles are heavy and, as a result, do not flow properly, as shown in Fig. 8. This improper flow adversely affects the reaction performance by reducing the amount of solar radiation reaching the particles. In addition, the particles create a structure like a fixed bed, which rests above the tube responsible for supplying air to the reaction mixture, affecting the dispersion of the air and creating preferential trajectories, resulting in a less efficient process. Furthermore, the aggregation of the photosensitizer on the silica particles can decrease the availability of active sites and hinder the efficient generation of ^1^O_2_, a crucial reactive species in the photocatalytic process (Kuznetsova 2012).

Chitosan immobilized carriers, unlike silica, are easy to handle even when moist. The spongin-immobilized carriers lie between silica and chitosan in terms of handling characteristics. Of the three materials studied, the spongin is the most expensive. Due to its geometry and liquid absorption capacity, it cannot fluidize. However, their porosity and fibrous structure remain constantly in contact with the airflow.

Concerning hydrodynamic behavior, chitosan demonstrates the best performance among the three carriers studied. The fluidization of chitosan immobilized carriers allows for greater contact between them, the airflow, and the wastewater, improving the oxygen singlet production and further degradation of organic matter in the effluent.

### Photolysis

Sunlight plays a crucial role in the photolysis process and is essential to access the sunlight effect in wastewater treatment. As the UV radiation in the sunlight can act as a selective reducer of organic compounds (Jing and Cao 2012), the lab-scale reactor was filled with 150 mL wastewater exposed to direct sunlight for 2 h, with an average irradiation of 715.55 ± 55.75 W/m^2^. During this assay, both wastewaters, E1 and E2, were evaluated.

**Fig. 9:**
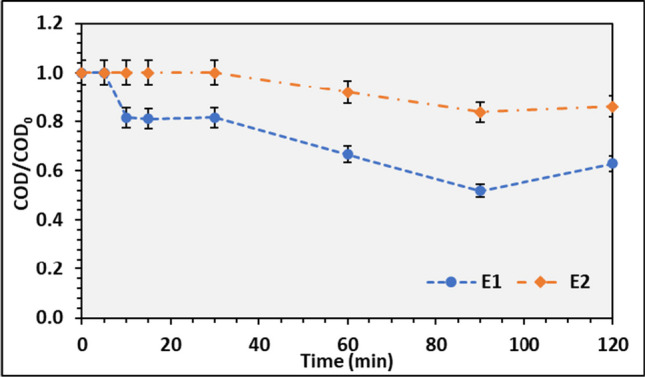
COD/COD_0_ average ratio and respective standard deviation during the photolysis assays for E1 and E2 wastewaters

Figure 9 presents the ratio COD/COD_0_ during the photolysis assay for both E1 and E2 wastewaters. In the first 30 min of the experiment, there was no significant variation in COD degradation for both E1 and E2 wastewater; however, after 30 min, a noticeable decrease in the COD ratio was observed, which may be related to the UV action in the wastewater. These results are like those reported by Jing and Cao (2012). Based on the observation that within 30 min of the experiment, the COD removal using UV light was low, and a hydraulic retention time (HRT) of 30 min was defined for further photooxidation experiments.

The COD degradation during the photolysis assay can be related to its sunlight absorption, as the dissolved organic matter (DOM) can act as a photosensitizer in the aquatic medium. DOM can occur naturally in waterbodies or added from wastewater discharges. Where the DOM from wastewater produces triplet excited states more efficiently, promoting indirect photodegradation (Sardana et al. 2022). This fact contributes to COD reduction during photolysis, reaching COD removals of 37% and 14% for wastewater E1 and E2, respectively.

### Carrier adsorption

The materials used as carriers are porous solids, allowing the immobilization of the photosensitizer by adsorption. This experiment was performed in the dark to ensure the action of the PS by itself. The COD was measured initially and during the 2 h of the assay.

**Fig. 10:**
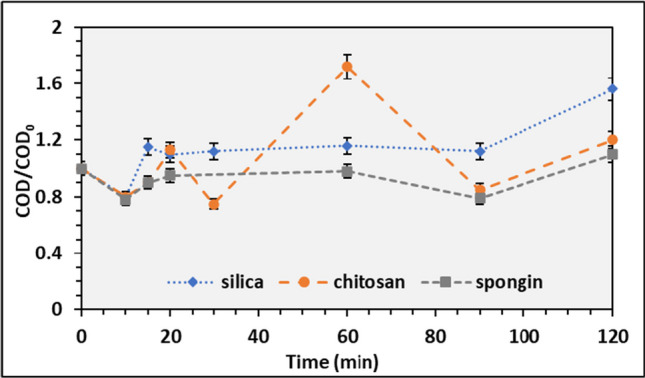
Average COD_0_/COD ratio of adsorption and respective standard in silica, chitosan, and spongin IC

There is no significant change in the COD value during the reaction time for silica, chitosan, and spongin. However, the chitosan presents more fluctuation during the experiment, as seen in Fig. 10.

The COD value does not present any reduction but a slight increase due to the desorption of the PS in the aquatic medium. Lower reaction times are preferable, as demonstrated in Fig. 10. After 20 min, the COD/COD_0_ ratio is approximately 1; after this point, a change in the behavior is observed.

### Photooxidation

During the heterogeneous photooxidation process, all three carrier materials were used and wastewater E1 for silica IC and E2 for chitosan and sponging. The assays were performed in duplicates and spanned 120 min. The operational conditions for the photooxidation assays carried out with the immobilized carriers are presented in Table 3.

**Table 3:** Working parameters for experimental assays with silica, spongin, and chitosan IC

Test	Carrier material	Wastewater	Volume (mL)	IC mass (g)	pH	Average radiation (W.m^−2^)
A	Silica	E1	150	30.0	8.00	771.3
B						744.4
C	Spongin	E2	150	1.00	8.00	685.0
D					12.0	582.6
E	Chitosan	E2	150	20	8.00	603.6
F					12.0	752.85
G				25	8	598.8

**Fig. 11:**
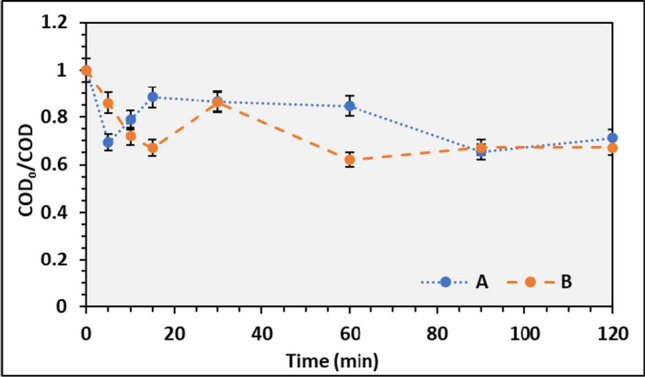
Average COD_0_/COD ratio and respective standard deviation during the photooxidation for silica IC, performed on different days

Several parameters in the reaction medium, such as pH and PS concentration, affect the photooxidation process (Kuznetsova 2012). In the initial assays, silica was used as the immobilized carrier (IC) to assess its photooxidation capacity. The wastewater used in assays A and B was the E1. From Fig. 11, one can observe a reduction in COD. However, when comparing these results with the photolysis assay, it is possible to conclude that the COD reduction at the end of the assay is similar: approximately 25 to 35% of the initial COD was removed in both assays. Suggesting that the poor hydrodynamics performance of silica strongly affects the photooxidation process, resulting in lower COD removal compared to the photolysis assay. Therefore, silica should not be used as IC for the photooxidation process due to its unfavorable hydrodynamics behavior. Neves et al. (2019) and Ronzani et al. (2013) reported using silica gel as a carrier material. The removal of metoprolol was effective with using porphyrin as a PS, reaching 60% (Neves et al. 2019).

Similarly, in the Ronzani et al. (2013) study, the removal of α-terpinene was successfully removed when a silica-immobilized photocatalyst was used. However, the silica used in this study was commercialized silica gel beads, which had a high density, contrary to the silica gel material reported. Promoting the formation of a compacted bed at the bottom of the reactor, as shown in Fig. 8a, impacts the air distribution and, consequently, the ^1^O_2_ production.

The assays performed with spongin and chitosan were achieved with wastewater E2 under different pH values, 8 and 12, as some studies reported that higher pH values increase the PS lifetime in the triplet state (Gerdes et al. 1997; Ozoemena et al. 2001; Potter et al. 1975). The pH adjustment was achieved by adding NaOH solution (0.1 N) to the wastewater.

**Fig. 12:**
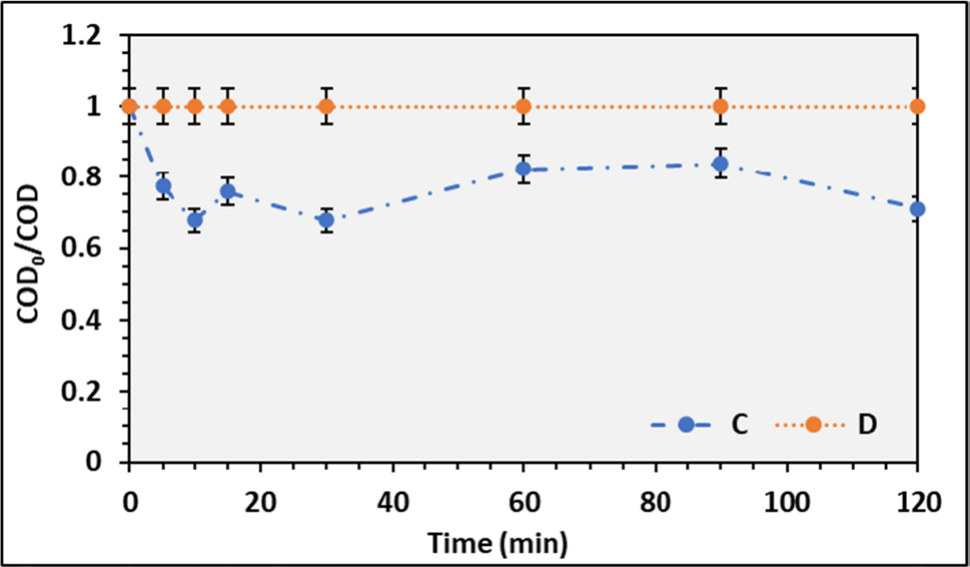
Average COD_0_/COD ratio and respective standard deviation for photooxidation using spongin IC at different pH working conditions. C, 8 and D, 12

Figure 12 presents the photooxidation spongin IC results at different pH values. One can observe that the increase in the pH does not result in any COD reduction, suggesting that no reaction happens during the assay. Compared to those obtained at pH = 8, the COD removal was approximately 30%, twice the one reached during the photolysis assay for the E2 wastewater, which was 15%, indicating that photooxidation occurred. However, by observing Fig. 8c, it can be seen that no reduction of COD occurs, certainly due to aggregation of the chitosan at the top of the reactor, which reduces the ^1^O_2_ production (Kuznetsova 2012). The use of sponge as a carrier material was evaluated by (Norman et al. 2018), where spongin was used as a carrier to support iron phthalocyanine to remove halophenols and bisphenol A, showing to be effective in removing all the pollutants present in the wastewater. However, the phenol removal was evaluated, and the COD removal efficiency was not yet accessed. The authors concluded that the spongin IC is not effective for the aim of the work.

**Fig. 13:**
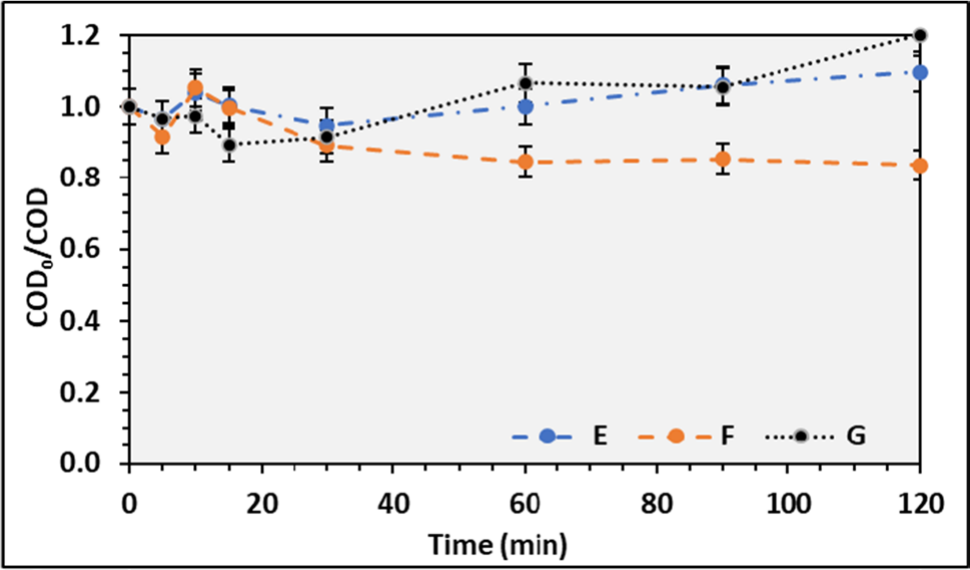
Average COD_0_/COD ratio and respective standard deviation for photooxidation using chitosan IC at different working conditions. E, pH = 8; F, pH = 12; and IC mass. E and F with 20 g of IC and G with 25 g of IC at pH = 8

Regarding the assays performed with chitosan, besides the pH effect, the mass of IC was also evaluated. By observing Fig. 13, it is evident that a higher removal efficiency of COD can be achieved for pH = 12, 20%, compared to pH = 8, where the final COD was higher than the initial concentration. By analyzing the IC mass, the COD removal efficiency decreased when it was increased, and the last COD was higher than at the beginning of the experiment. This trend is like the experiments conducted with pH = 8. The COD increment at the end of the experiment can be explained by the PS bleaching from the carrier to the reactional medium, like the results observed in Fig. 10, during the adsorption assay. Overall, the photooxidation process reaches the maximum COD reduction in the first 15 to 30 min of reaction, concluding that shorter reactional HRT is preferable. Equally to the other materials, chitosan as a carrier material for PS has also been reported. However, a successful removal is written just for phenol and parabens removals, with a high rate of carrier reuse (Foszpańczyk et al. 2019; Gmurek et al. 2017a, b). Leading to the conclusion that chitosan can be used as an IC and reused several times without losing its properties. Previous studies of the authors show that COD reduction in homogeneous operational conditions is possible (Santos et al. 2023); nevertheless, further assays must be addressed to reach the optimal working conditions to promote an effective COD reduction in those wastewaters.

## Conclusions

The success of the immobilization process was unmistakably affirmed through a noticeable change in the color of the carrier material, attributable to the presence of ZnPcSO4 with its distinctive blue hue. Various analyses, including FTIR, XRD, BET, and SEM, validated the photosensitizer’s presence.

FTIR analysis showed increased absorption in the 3600–3100 cm^−1^ range, indicating the presence of O–H and N–H groups important for immobilization. XRD analysis confirmed an amorphous structure in the material. BET analysis revealed a reduced surface area, suggesting the photosensitizer occupied carrier material pores.

Chitosan was the most effective carrier for hydrodynamic behavior, enhancing singlet oxygen production and organic matter degradation. However, the materials increased COD values due to photosensitizer desorption. Chitosan outperformed the other carriers in reducing COD. To enhance the photooxidation process, efforts should focus on reducing photosensitizer bleaching and optimizing pH values for continuous operation.

## Data Availability

The data that support the findings of this study are available from the corresponding author upon reasonable request.
